# Folate Receptor Beta for Macrophage Imaging in Rheumatoid Arthritis

**DOI:** 10.3389/fimmu.2022.819163

**Published:** 2022-02-02

**Authors:** Maarten M. Steinz, Aiarpi Ezdoglian, Fatemeh Khodadust, Carla F. M. Molthoff, Madduri Srinivasarao, Philip S. Low, Gerben J. C. Zwezerijnen, Maqsood Yaqub, Wissam Beaino, Albert D. Windhorst, Sander W. Tas, Gerrit Jansen, Conny J. van der Laken

**Affiliations:** ^1^ Department of Rheumatology and Clinical Immunology, Amsterdam University Medical Center, VU University Medical Center (VUmc), Amsterdam, Netherlands; ^2^ Department of Radiology & Nuclear Medicine, Amsterdam University Medical Center, VU, Amsterdam, Netherlands; ^3^ Department of Chemistry, Purdue University, West Lafayette, IN, United States; ^4^ Department of Rheumatology and Clinical Immunology, Amsterdam University Medical Center, AMC, Amsterdam, Netherlands

**Keywords:** PET imaging, folate receptor beta, rheumatoid arthritis, macrophage, antigen-induced arthritis

## Abstract

Non-invasive imaging modalities constitute an increasingly important tool in diagnostic and therapy response monitoring of patients with autoimmune diseases, including rheumatoid arthritis (RA). In particular, macrophage imaging with positron emission tomography (PET) using novel radiotracers based on differential expression of plasma membrane proteins and functioning of cellular processes may be suited for this. Over the past decade, selective expression of folate receptor β (FRβ), a glycosylphosphatidylinositol-anchored plasma membrane protein, on myeloid cells has emerged as an attractive target for macrophage imaging by exploiting the high binding affinity of folate-based PET tracers. This work discusses molecular, biochemical and functional properties of FRβ, describes the preclinical development of a folate-PET tracer and the evaluation of this tracer in a translational model of arthritis for diagnostics and therapy-response monitoring, and finally the first clinical application of the folate-PET tracer in RA patients with active disease. Consequently, folate-based PET tracers hold great promise for macrophage imaging in a variety of (chronic) inflammatory (autoimmune) diseases beyond RA.

## 1 The Role of Folate Receptor Beta For PET Imaging in Arthritis

### 1.1 Synovial Macrophages as Biomarkers for RA Disease Activity Assessment

Rheumatoid arthritis (RA) is an autoimmune disease of the joints characterized by the infiltration of various immune cells in the synovium amongst which macrophages play an important role (1, 2). Macrophages impact on other immune cells and inflammatory processes *via* the release of proinflammatory cytokines (e.g. TNFα) and chemokines, which may promote activation of T cells and other immune cells, trigger endothelial cell activation and (pathological) angiogenesis, and induce osteoclast activation (3, 4). Synovial tissue analysis has pointed out that the (activated) synovial macrophage is a key biomarker for disease activity assessment from the early disease onwards and for monitoring of therapeutic efficacy at later stages of the disease (5, 6). The basic synovial joint architecture of the healthy joint is comprised of a double layered structure (synovial lining) which holds tissue resident macrophages, and underneath a vascularized sub-lining layer of connective tissue (7). In the early stages of RA, infiltration of immune cells is observed in combination with activation of resident macrophages present in the synovial lining layer (2). Established RA is marked by progressive macrophage infiltration in the synovium (~10-20 layers) (8). In fact, macrophages represent one of the most prominent cell types present in the synovium during early stage and also established RA (2, 9, 10), being responsive to treatment (6), and thus underscoring their exploitation as a biomarker for the assessment of RA disease through positron emission tomography (PET) imaging.

The importance of macrophages as key player in the pathogenesis of RA has been explored in both preclinical and clinical studies. It has for example been shown in animal models of arthritis that depletion of macrophages significantly decreases the severity of the disease (11, 12). Also, in RA patients, macrophage infiltration in the RA synovium has been found to significantly correlate with disease severity (e.g. with changes in disease activity score (DAS) and composite change index) (5, 6, 13). In addition, recent in depth cellular and molecular analyses of RA synovial tissues revealed that RA patients could be stratified in 3 pathology groups based on the presence of specific immune cell types (14–16). These 3 pathotypes were designated *diffuse-myeloid* (characterized by predominantly myeloid cell infiltration, notably macrophages), *lympho-myeloid* (predominantly B-cell infiltration), and *pauci-immune* (low immune cell infiltration) (14). Remarkably, a higher diffuse-myeloid gene expression profile, thus characterized by macrophage infiltration, was associated with a higher DAS 28-ESR and a larger DAS 28-ESR reduction after treatment with disease modifying anti-rheumatic drugs (DMARDs) (14), again underscoring the importance of the synovial macrophage as biomarker for RA disease activity.

Although it is well recognized that macrophages play an important role in the pathology of RA, they can exert both pro- and anti-inflammatory roles associated with their polarization (17). The synovial cytokine milieu, in particular granulocyte-macrophage colony-stimulating factor (GM-CSF) and macrophage colony-stimulating-factor (M-CSF), constitutes the driving force in skewing macrophages to the M1-type pro-inflammatory phenotype and the M2-type anti-inflammatory macrophage, respectively (9, 18–20). M1 and M2 represent the extremes of macrophage polarization and have been characterized based on differences in their transcriptome, secretome and proteome profiles (19–22). Several (membrane) marker proteins are commonly used to classify M1 (e.g. CD80, TNFα, iNOS) and M2 (e.g. CD163, IL-10, Arginase) macrophage subpopulations, and have been associated to an inflammatory or remission state of RA. For example, macrophage subpopulations were found associated with RA disease remission such as synovial tissue macrophages that are MerTK positive (MerTKpos), lymphatic vessel endothelial hyaluronan receptor 1 positive (LYVE1pos) and have a high expression of Folate Receptor (FR) beta (FRβ-high) (23). In the context of this review, FRβ expression has long been recognized on macrophages that are triggered by inflammatory stimuli and on activated macrophages in inflamed joints of RA patients (24, 25). In ex-vivo M-CSF skewed monocyte-derived macrophages, FRβ is differentially expressed on M2-type macrophages (26–28). However, in inflamed RA synovium, these FRβ-expressing M2-macrophages can produce pro-inflammatory cytokines when exposed to either pro-inflammatory stimuli (i.e. lipo-polysaccharide (LPS) + interferon-γ (IFNγ) (19) or an RA synovial microenvironment with anti-citrullinated protein antibodies or complex IgGs (29, 30). Together, FRβ is a bona fide marker on synovial macrophage subpopulations, even though its exact role in function in either pro- and anti-inflammatory macrophages needs to be defined in greater detail. FRβ expression on (activated) macrophages in RA has initiated research aimed at therapeutic targeting as well disease monitoring with imaging modalities (31, 32), which will be discussed in the next sections.

### 1.2 Folate Receptors: Function, Structure and Targeting

**Figure 1:**
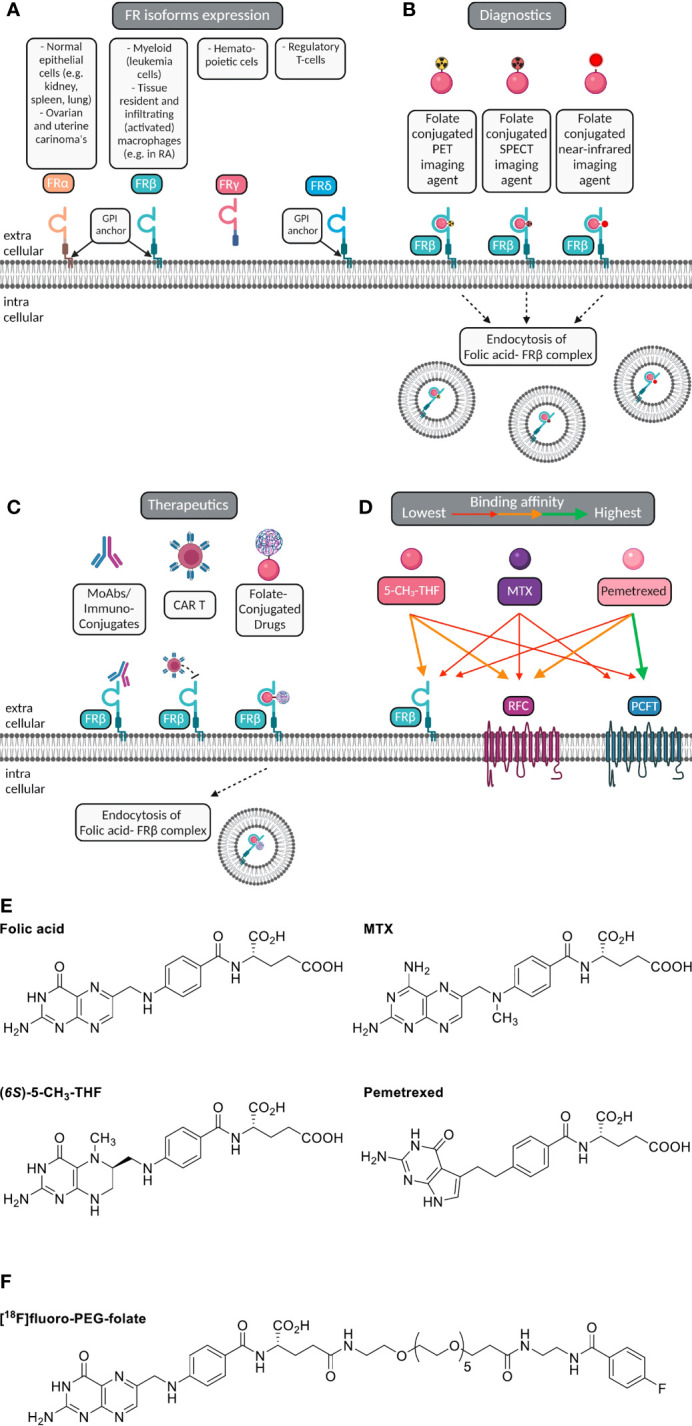
Macrophage FRβ for folate-based imaging and therapeutic targeting. **(A)** Folate receptor isoforms FRα, FRβ, FRγ and FRδ, their GPI-membrane anchoring (except FRγ), and cell/tissue expression. **(B)** Selective expression of FRβ (cyanin), a glycosylphosphatidylinositol-anchored plasma membrane protein, on myeloid cells (e.g. macrophages) constitutes a suitable target for imaging of inflammatory disease, including RA. Folate (pink ellipse) is coupled to a radioactive isotope (for PET or SPECT) or near infrared fluorescent dye (for optical imaging). Following high affinity binding to FRβ, these imaging agents may stay membrane bound or potentially internalized *via* endocytosis. **(C)** Macrophage FRβ can also be subject to therapeutic targeting to ameliorate inflammation. This can be achieved by (drug-conjugated) monoclonal antibodies, folate-conjugated drugs, CAR-T cells targeted towards FRβ and with small molecule folate antagonists. **(D)** Folic acid, the primary circulating plasma folate form 5-CH_3_-THF, and folate antagonist therapeutic drugs MTX or PMX can bind to FRβ or one of the two other folate carriers expressed on macrophages, i.e. RFC/SLC19A1 and PCFT/SLC46A1. The binding affinity of 5-CH_3_-THF, MTX and PMX varies for FRβ, RFC and PCFT, respectively, as indicated by colored arrows. For example, 5-CH_3_-THF binding affinity to FRβ > MTX and PMX. MTX transport is facilitated by all three folate carriers, but with slightly higher affinities for RFC and PCFT. PMX displays the highest affinity to PCFT, moderate affinity to RFC and the lowest affinity for FRβ. **(E)** Chemical structures of folic acid, (*6S*)-5CH_3_-THF, methotrexate (MTX) and pemetrexed (PMX) illustrating the shared pterin moiety which is captured in the folic acid binding cleft of FRβ. **(F)** Chemical structure of [^18^F]fluoro-PEG-Folate, the folate PET imaging agent for high-affinity binding to FRβ on macrophages and utilization for diagnostics and therapy response monitoring.

Human FRs are high affinity binding proteins for folates (folic acid and reduced folate cofactors) which are essential vitamins necessary for single carbon transfer reactions in amino acid biosynthesis (e.g. conversion of homocysteine into methionine) and for *de novo* purine and thymidylate biosynthesis (33–35). There are four types of FRs: FR-alpha (FRα), FR-beta (FRβ), FR-gamma (FRγ) and FR-delta (FRδ) (**Figure 1A**). Of those, FRα, FRβ and FRδ are glycosylphosphatidylinositol (GPI) membrane anchored, whereas FRγ is a secreted form (mainly from hematopoietic cells) because of a lack of an efficient signal for GPI modification (36–39). FRα is expressed on normal epithelial cells (e.g. kidney, spleen and lung tissue) (40) and tumor tissues like ovarian, breast (41), pancreatic and lung carcinomas (42, 43). FRβ is selectively expressed on cells of the myeloid lineage (44, 45) and is upregulated on activated macrophages in active RA disease (24, 25) wherein its expression is regulated by PU.1 transcription factor (46). The FRδ gene was originally identified being highly homologous mouse folate binding protein 3 (Folbp3) (47), but does not harbor folate binding capacity (48). FRδ (and splice variants thereof) is expressed on regulatory T cells and has a proposed function in immune regulation (49).

Macrophage FRβ is a valid target for folate-based imaging (**Figure 1B**), along with other FRβ targeting approaches (**Figure 1C**) by (drug-conjugated) monoclonal antibodies, folate-conjugated drugs, CAR T cells and folate antagonists (25, 31, 34, 50, 51). FRβ contains a binding pocket for folic acid where binding of folic acid is facilitated by a conformational change in two regions of the receptor, in particular the region connecting beta strand 1 and 2 and the region of alpha helix 1 (52). Like folic acid, folate antagonist therapeutic drugs such as methotrexate (MTX, the anchor drug in RA treatment) (53, 54) and pemetrexed (PMX) (55) share a pterin moiety in their structure which can bind in the hydrophobic region of the binding pocket of FRβ (52) (**Figure 1D**). FRα and FRβ share high affinity binding of folic acid with Kd’s in the low nanomolar range (0.1-1 nM) (36). Structure activity testing disclosed anti-folate structures with FRα and FRβ affinities close to folic acid, 2-3 orders of magnitude lower affinities for MTX than folic acid and intermediate affinities for PMX (25, 56).

In order to elicit therapeutic activity against macrophages, a folate antagonist should compete with FRβ binding of the primary circulating reduced folate in plasma, 5-methyltetrahydrofolate (5-CH_3_-THF) (25). Also FRβ functions as one of three transport proteins for folates and antifolates, the two others are the reduced folate carrier (RFC, SLC19A1) and proton-coupled folate transporter (PCFT, SLC46A1) (57) (**Figure 1E**). The expression of these 3 folate transporters does vary between polarized macrophages; in *ex vivo* skewed monocytes to M1-type macrophages by GM-CSF and M2-type macrophage by M-CSF, RFC gene expression is differentially higher in M1-type macrophages, whereas FRβ and PCFT expression are markedly higher in M2-type macrophages (27, 28). Given that FRα/β also retain a high affinity binding profile for folate conjugates, this allowed the design of folate conjugates which could serve as folate based imaging agents like [^18^F]fluoro-PEG-folate (**Figure 1F**) (58–60).

### 1.3 PET Imaging of Macrophages

Over the past decade, non-invasive molecular imaging techniques that assess RA disease activity using macrophage PET imaging have been developed (31, 58, 61–63). Disease activity assessment of RA is currently performed clinically through calculation of the DAS, which takes into account the number of swollen and tender joints, the erythrocyte sedimentation rate (ESR) and the visual analogue score (VAS) (1). However, the DAS score contains subjective elements and is limited to the sensitivity and specificity of clinical assessment of tenderness and swelling of the joints (64). To make further steps in improvement of diagnostics and monitoring of disease activity, objective tools such as macrophage (whole body) PET imaging may offer new opportunities for early diagnosis and early determination of the treatment outcome. Early diagnosis (even before clinical diagnosis) and effective, personalized treatment may ultimately result in prevention of (progression of) joint damage (1).

Initially, imaging studies were performed for FRα-expressing tumors with folate-SPECT tracer [^99^Tc]EC20 (48). By serendipity, imaging of a cancer patient who had an arthritic comorbidity showed a positive scan of the inflamed knee joint due to infiltration of FRβ-positive macrophages (65). This was the start of exploration of arthritis imaging by FRβ targeting. The easy accessibility as GPI-linked plasma membrane protein and its myeloid cell specific expression constitutes FRβ a suitable target for macrophage imaging. [^99^Tc]EC20 proved its suitability in visualizing FRβ-positive macrophages in inflamed joints of arthritic rats (66) and RA patients (67). These findings encouraged the development of folate-based PET tracer which would provide a higher sensitivity and spatial resolution compared to scintigraphy.

The original synthesis of folate-based PET tracers relied on chemistry linking folic acid with an spacer moiety (polyethylene glycol) as a precursor molecule to which the PET isotope is coupled (68). This approach was adopted in studies by Gent et al. (58) and Kularatne et al. (59) to synthesize [^18^F]fluoro-PEG-folate (**Figure 1F**). Assessment of FR binding affinity for fluoro-PEG-folate in a [^3^H]folic acid competition assay showed that the unlabeled tracer had a 2-fold lower affinity than for folic acid, but a 2.5-fold higher affinity than for the circulating plasma folate 5CH_3_-THF (58). After fulfilling this important criterium of high affinity binding and outcompeting binding of circulating plasma 5-CH_3_-THF, next [^18^F]fluoro-PEG-folate was examined further for *in vivo* PET–based monitoring of disease activity and therapy response in a preclinical model of RA (see section 2).

## 2 Folate Receptor β-Targeted Imaging in the Antigen-Induced Arthritis Model

### 2.1 The Antigen-Induced Arthritis Model

**Figure 2:**
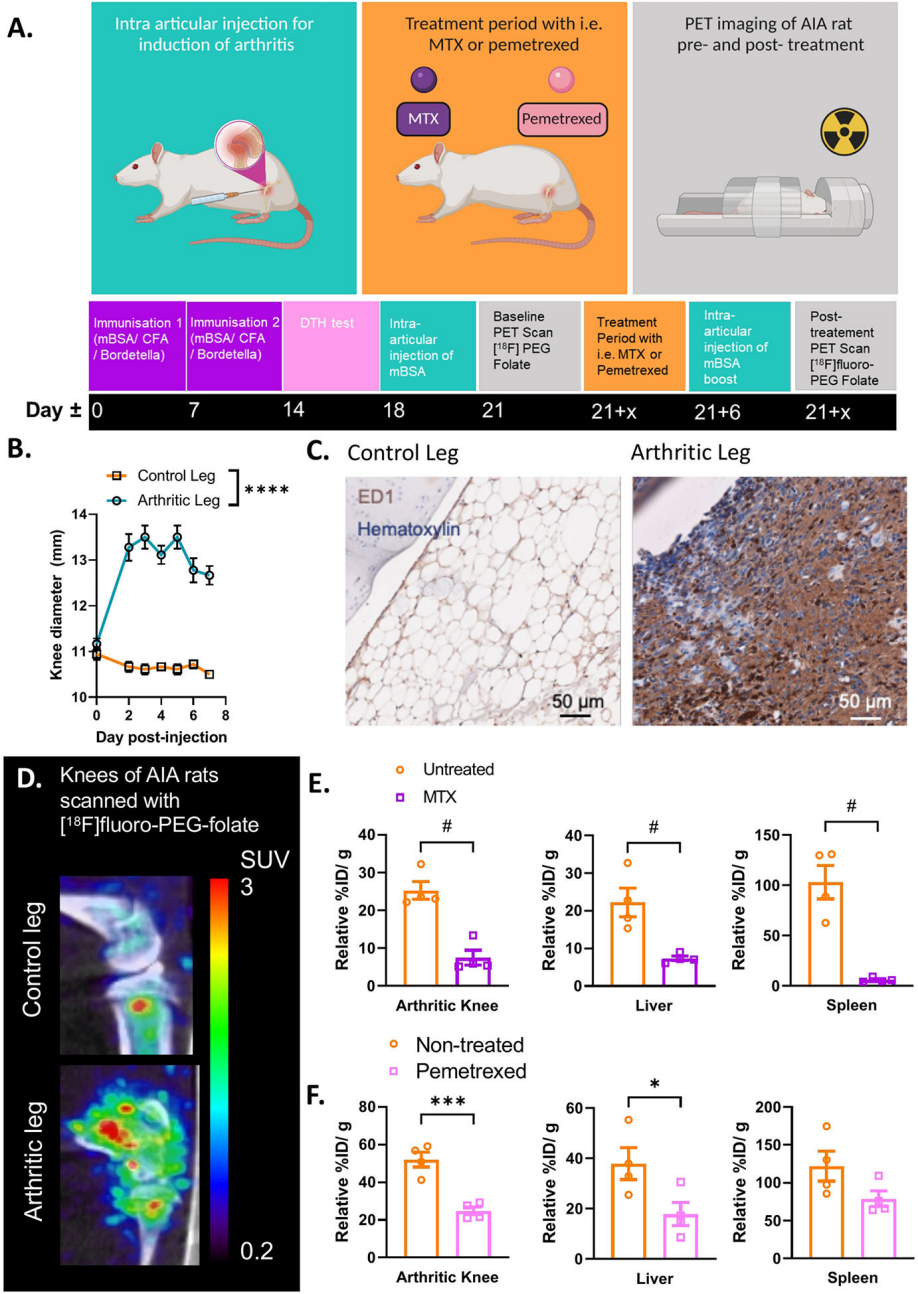
Antigen induced arthritis (AIA) rat model used in preclinical studies for PET imaging with [^18^F]fluoro-PEG-folate tracer. **(A)** Schematic representation of the preclinical set up of the experiment: starting at day 0 and day 7 with an immunization of the rat with an emulsion of Complete Freund’s Adjuvant, Custom Bordetella Pertussis and methylated BSA (mBSA). Immunization status is checked at day 14 with a delayed type hypersensitivity (DTH) test through injection of mBSA in the ear. Around day 18, an intra-articular (i.a.) injection is given in one of the knees with mBSA. During the whole duration of the experiment or after i.a. injection, the rat can be treated with FRβ-targeted folate antagonist such as MTX or PMX. A baseline (/pre-treatment) PET scan and post-treatment PET scan with [^18^F]fluoro-PEG-folate have been used to monitor the effect of antifolate therapy. **(B)** After 1-3 days post i.a. injection a significant swelling of the knee diameter is seen in the arthritis-affected leg (****p < 0.0001, two-way ANOVA, N = 9 rats/group, paired samples). **(C)** Representative image of macrophage infiltration in the synovium of the arthritic leg as detected by immunohistochemical (IHC) DAB-staining of rat knee tissue with ED-1 antibody (HM3029, Hycult Biotech) (scale bar = 50μm). **(D)** Illustrative image of increased [^18^F]fluoro-PEG-folate uptake in the arthritic knee (lower panel, arthritic leg) vs. the non-arthritic, contra-lateral knee (upper panel, control leg) of an AIA rat. Both images are scaled to the same standard uptake value based on the injected dose (in MBq/ml) of the tracer and the body weight of the animal (in g.). **(E)** Biodistribution of [^18^F]fluoro-PEG-folate in the arthritic knee, liver and spleen of non-treated and MTX-treated AIA rats. Data were corrected for blood %ID/g. **(F)** Biodistribution of [^18^F]fluoro-PEG-folate in the arthritic knee, liver and spleen of non-treated and PMX-treated AIA rats. Data were corrected for blood %ID/g. Statistics for images E-F were performed in Graph-Pad Prism version 9, ^#^p < 0.01, Mann-Whitney U test for non-parametric divided data, *p < 0.05, ***p < 0.001, unpaired T-TEST for parametric divided data, N = 4 rats/group. All results described in Figure 2 were derived (and reanalyzed were indicated) from own research (58, 62, 69, 70).

In the preclinical assessment of FRβ as a macrophage target for PET imaging of arthritis, Chandrupatla et al. established a pre-clinical rat model of arthritis with sustained macrophage infiltration in the joints (69). In this model arthritis is induced through immunization with methylated bovine serum albumin (mBSA) in complete Freund’s adjuvant (CFA) and custom Bordetella pertussis antigen (CBP) following one or repeated intra-articular (i.a.) injections with mBSA in one knee of the rat (leaving the contralateral knee as control), after which synovial inflammation accompanied by an increase in knee thickness occurs over 3 days (**Figures 2A, B**) (69). This antigen-induced arthritis model was selected for PET imaging of molecular markers in arthritis and therapeutic evaluations due to several reasons; (i) the model is mono-articular, and hence the contra-lateral and other joints can serve as control, (ii) the model is relatively mild and is not accompanied by severe bone destruction or polyarticular involvement, (iii) the model is also reminiscent of human RA featuring macrophage infiltration (71, 72) (**Figure 2C**) and moderate systemic inflammation manifested by modest macrophage infiltration in the liver and spleen (70), (iv) macrophage infiltration can be sustained by repeated mBSA injections, which is a good condition for therapeutic monitoring (69), and (v) the larger size of the rat allows injecting more radioactivity and the larger knee size of the rat (compared to a mouse) is advantageous given the spatial resolution of most PET scanners. This model therefore allows for studying if [^18^F]fluoro-PEG-folate is a suitable tracer for objective macrophage imaging in arthritis affected joints and for longitudinally studying the effect of anti-rheumatic drugs on joint macrophage infiltration (58, 62, 70).

### 2.2 FRβ-Targeted Macrophage PET Imaging in Arthritic Rats

Initially, imaging for FRβ in preclinical arthritis models and RA patients was performed with the SPECT tracer [^99m^Tc]EC20 (66, 67). It has only been up until recently that the [^18^F]-PEG-folate tracer has been tested pre-clinically in a model of arthritis (i.e. the AIA model). Gent et al. showed that in arthritic rats scanned with [^18^F]fluoro-PEG-folate had a ~50% increased tracer uptake in the arthritic knee compared to the contralateral uninflamed knee joint (58) (see illustrative image **Figure 2D**). Gent et al. also showed that the [^18^F]fluoro-PEG-folate tracer specifically targeted folate receptors since blocking of this receptor with unlabeled glucosamine-folate significantly abolished [^18^F]fluoro-PEG-folate uptake in the arthritic knees (58).

Treatment of arthritic rats with different dosages of the folate antagonist MTX significantly reduced (~2-4 fold) tracer uptake in the arthritic knee compared to the arthritic knee of untreated rats (62, 70) (**Figure 2E**). Consistently, histological analysis demonstrated that macrophage infiltration was also ~2-4 fold reduced in the arthritic knee as well as in the liver and spleen following MTX therapy (70). Involvement of liver and spleen point to systemic inflammation in the arthritic rat model, which is suppressed by MTX treatment (70). This is in line with the systemic character of RA in patients (73). Similar results were obtained after treatment of arthritic rats with the second-generation folate antagonist pemetrexed (PMX)/Alimta (74). [^18^F]fluoro-PEG-folate uptake in the arthritic knee, liver and spleen of PMX treated rats was reduced ~2.2; 3.2 and 1.6-fold, respectively compared to untreated rats (**Figure 2F**). PMX was originally developed to overcome MTX resistance in cancer chemotherapy (55) by harboring more efficient transport properties *via* RFC, PCFT and FR than MTX (**Figure 1C**). PMX has shown anti-arthritic effects by suppressing cytokine production in activated T cells of RA patients (75), experimental arthritis models (74) and polarized macrophages *in vitro* (28). Together, these preclinical studies demonstrate the suitability and feasibility of macrophage PET with [^18^F]fluoro-PEG-folate for arthritis disease monitoring and therapy response monitoring thereby supporting clinical studies in RA. Preclinical animal studies also revealed that [^18^F]fluoro-PEG-folate may provide improved arthritis imaging compared to an established macrophage translocator protein (TSPO) tracer *(R)*-[^11^C]PK11195 (58) as a 2.3-fold higher arthritic knee over blood ratios was observed for [^18^F]fluoro-PEG-folate than for (*R*)-[^11^C]PK11195.

## 3 Clinical Evaluation of Folate PET Imaging in RA Patients

### 3.1 First Clinical Study With [^18^F]fluoro-PEG-Folate in RA Patients

**Figure 3:**
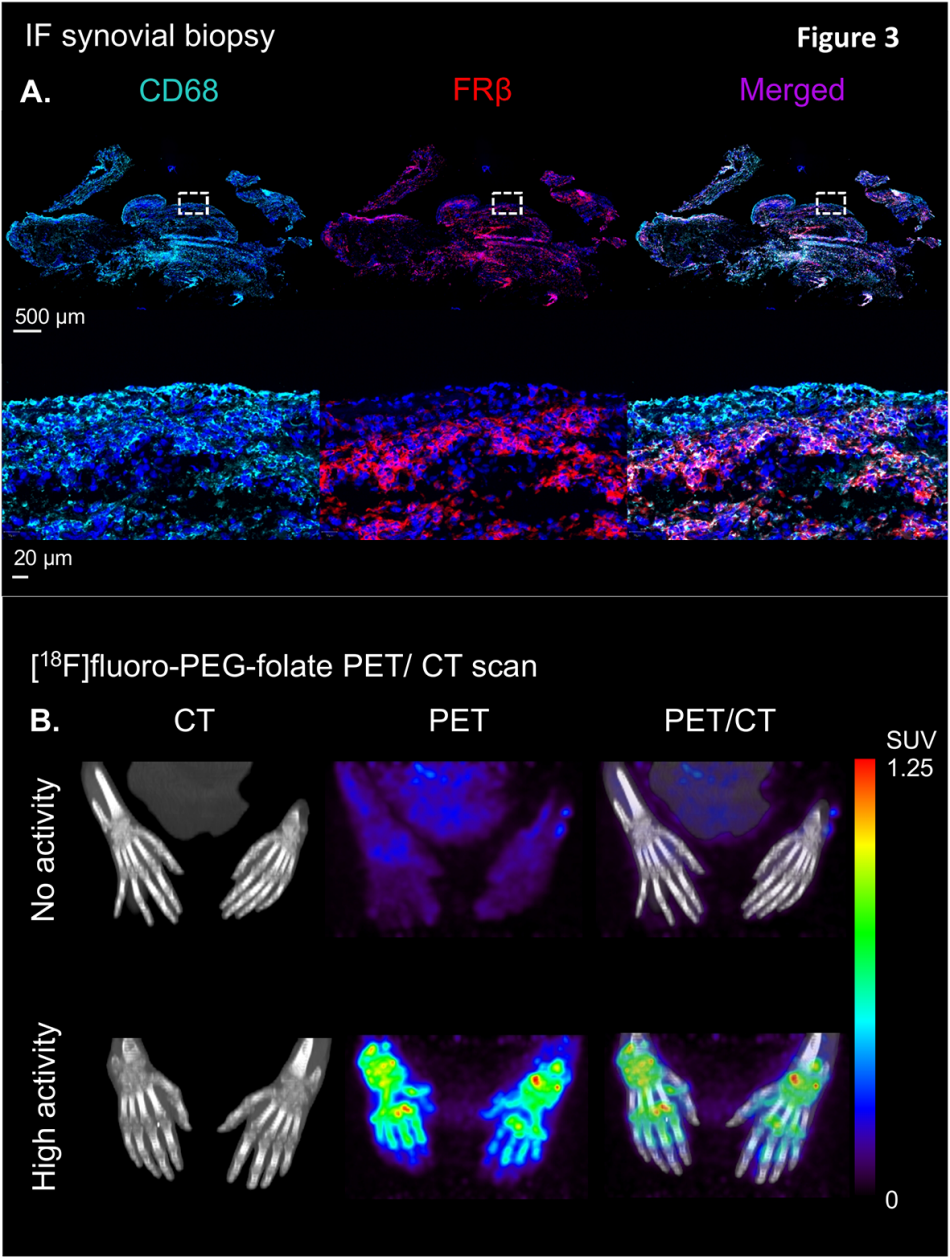
Illustration of the clinical validation of the [^18^F]fluoro-PEG-folate tracer in patients with rheumatoid arthritis. **(A)** *Upper panel* showing an illustrative example of immunofluorescent staining of CD68 (left), FRβ (middle) and CD68 + FRβ merged staining in a synovial knee biopsy of an RA patient ​with active disease. *Lower panel* depicts marked expression of CD68- and FRβ-positive macrophages in both synovial lining and sublining (see the zoomed view of the dashed marked box in the upper panel of the RA synovial tissue. **(B)** Representative example of high specific uptake of [^18^F]fluoro-PEG-folate in the hand joints of a patient with high RA disease activity vs. marginal uptake in the hand joints of a RA patient with low disease activity in the hands. Both images are scaled to the same standard uptake value based on the injected dose (in MBq/ml) of the tracer and the body weight of the patient (in kg.). All representative images of Figure 3 were derived from own research (25, 61).

Immuno-histochemical and immuno-fluorescent analysis of FRβ expression in RA synovial biopsies showed marked expression of double CD68 and FRβ-positive macrophages in both lining and sublining of RA synovial tissue (representative example shown in **Figure 3A**) (25), thus encouraging further clinical evaluation of the [^18^F]fluoro-PEG-folate PET tracer. The first clinical evaluation of the [^18^F]fluoro-PEG-folate PET tracer for arthritis was performed by Verweij et al. in RA patients (61). This study showed uptake of the [^18^F]fluoro PEG folate PET tracer in clinically active joints (**Figure 3B**). Although the absolute tracer uptake of [^18^F]fluoro-PEG-folate in arthritic joints was ~ 2.5-fold lower than for the previously investigated macrophage tracer (*R*)-[^11^C]-PK11195, the target-to-background ratios of [^18^F]fluoro-PEG-folate PET-CT were significantly higher (3.5 ± 2.2 versus 1.7 ± 0.6; p<0.02; n=6 patients) (61). This was a relevant improvement regarding clinical application of macrophage PET imaging in RA, since PK11195 (first generation TSPO) imaging in RA is limited by relatively high background uptake in bone marrow and peri-articular tissues (e.g. muscle) (76). As a consequence, more subtle arthritis activity can easily be missed, which is particularly relevant for early disease assessment and highly sensitive monitoring of therapeutic efficacy. Both clinically and sub-clinically inflamed joints were imaged by folate PET-CT with lower false positive and false negative findings (as compared to clinical findings) than PK11195 PET-CT (61). This holds promise for [^18^F]fluoro-PEG-folate PET-CT in terms of potential predictive value in clinical RA diagnosis and development of relapse in established disease, since previous studies with PK11195 PET-CT already demonstrated predictive value for these clinical applications (76, 77), which thus may be further improved using [^18^F]fluoro-PEG-folate whole body PET-CT. Our preliminary data also point at the potential [^18^F]fluoro-PEG-folate PET-CT to monitor treatment efficacy of anti-rheumatic drugs including of anti-folates (70). [^18^F]fluoro-PEG-folate binding affinity towards FRβ outweighs methotrexate by at least 2–3 orders of magnitude (26, 58). If, additionally, a safe time window of 7 days is applied between last methotrexate administration and [^18^F]fluoro-PEG-folate, no blockade of [^18^F]fluoro-PEG-folate binding in arthritic joints of RA patients by anti-folates (61).

### 3.2 Future Perspectives

Given the fact that macrophage PET imaging with FRβ has shown clinical feasibility in RA patients, future challenges will be to use folate PET tracers for detection of disease activity in early stage RA and monitoring/prediction of the therapy response of targeted synthetic or biological DMARDs in joints and other sides affected by systemic inflammation. Furthermore, many other inflammatory diseases with macrophage involvement, e.g. idiopathic pulmonary fibrosis, systemic lupus erythematosus, scleroderma, psoriasis, ulcerative colitis, Crohn’s disease (78, 79), giant cell arteritis (80), cardiovascular diseases (81, 82) and tumor associated macrophages in oncology (43) may benefit from folate PET imaging to detect and monitor disease activity. Recently, also folate PET imaging of lung macrophages in COVID-19 was advocated to identify patients at risk of a severe or even lethal disease (83). Steps forward in the folate-linker chemistry also allow for more rapid synthesis of alternative folate tracers from precursor molecules, e.g. [^18^F]folate-PEG-NOTA-Al (84), which are promising but warrant further (pre)clinical evaluation. Beyond PET tracers, there is also increasing interest in the development of folate near- infrared/optical imaging agents for FRβ targeting in oncology and inflammatory diseases (85, 86). Altogether, FRβ remains a reputable target for continued research and (pre)clinical testing of imaging and therapeutic agents in a wide range of pathological conditions.

## Author Contributions

MS, AE, GJ, and CL: conceptual design, drafting of the article, critical scientific revision and approval of final version. MS: design and scientific translation of the reviewed literature to all figures. MS, FK, CM, PL, GZ, MY, WB, AW, ST, GJ and CL: critical revision and approval of the final version. All authors contributed to the article and approved the submitted version.

## Funding

The work of this review was supported in part by grant from the Center for Translationational Molecular Medicine (CTMM TRACER), the Dutch Rheumatism Fund (ReumaNederland, NRF 09-01-404), Cancer Center Amsterdam, and EU-Marie Curie ARCAID program.

## Conflict of Interest

The authors declare that the research was conducted in the absence of any commercial or financial relationships that could be construed as a potential conflict of interest.

## Publisher’s Note

All claims expressed in this article are solely those of the authors and do not necessarily represent those of their affiliated organizations, or those of the publisher, the editors and the reviewers. Any product that may be evaluated in this article, or claim that may be made by its manufacturer, is not guaranteed or endorsed by the publisher.
